# Corrosion Behaviors of Q345R Steel at the Initial Stage in an Oxygen-Containing Aqueous Environment: Experiment and Modeling

**DOI:** 10.3390/ma11081462

**Published:** 2018-08-17

**Authors:** Longjun Chen, Junying Hu, Xiankang Zhong, Qiang Zhang, Yan Zheng, Zhi Zhang, Dezhi Zeng

**Affiliations:** 1 State Key Laboratory of Oil and Gas Reservoir Geology and Exploitation, School of Oil and Natural Gas Engineering, Southwest Petroleum University, Chengdu 610500, China; chenlongjun530@126.com (L.C.); zhangzhi@swpu.edu.cn (Z.Z.); zengdezhi@swpu.edu.cn (D.Z.); 2 Research Institute of Natural Gas Technology, Southwest Oil and Gas Field Company of PetroChina, Chengdu 610213, China; zh_qiang@petrochina.com.cn; 3 School of Mechanical Engineering, Southwest Petroleum University, Chengdu 610500, China; zhengyan@swpu.edu.cn

**Keywords:** oxygen corrosion, low alloy steel, modeling, mixed potential theory

## Abstract

The ingress of oxygen into pressure vessels used in oil & gas production and transportation could easily result in serious corrosion. In this work, the corrosion behaviors of Q345R steel at the initial stage in 1 wt.% NaCl solution were investigated using electrochemical techniques. The effects of oxygen concentration, temperature and pH on corrosion behaviors were discussed. Simultaneously, a numerical model based on the mixed potential theory was proposed. The results show that the proposed model accords well with the experimental data in the pH range from 9.0 to 5.0. In this pH range, the oxygen reduction reaction, H^+^ reduction, water reduction, and iron oxidation can be quantitatively analyzed using this model. However, this model shows a disagreement with the experimental data at lower pH. This can be attributed to the fact that actual area of reaction on the electrode is much smaller than the preset area due to the block effect resulted from hydrogen bubbles adsorbed on the electrode surface.

## 1. Introduction

Oxygen could ingress into the oil & gas production and transportation systems in many ways. For instance, the waterflood process, gas injection process, inhibitor injection process, as well as pipeline leakage, could result in the ingress of oxygen into tubing, casing, surface-injection pipelines and equipment [1,2,3]. Usually, the oxygen content in surface lines and equipment is quite high, for example, the oxygen content in surface injection lines for high pressure air injection can be as high as 21% [4]. Oxygen-containing aqueous environment is highly aggressive to iron and its alloys because oxygen is a strong oxidizer and it is able to increase the corrosion rate by increasing the cathodic reaction rate.

In the past decades, the corrosion related to oil & gas production and transportation such as CO_2_ corrosion [5,6], H_2_S corrosion [7,8], microbiologically induced corrosion [9,10], acetic acid [11], and naphthenic corrosion [12] has been extensively studied. Compared with these types of corrosion mentioned above, the oxygen corrosion could cause a threat at the same level or even higher to the lines and equipment used in oil & gas industry. On the one hand, it can cause serious corrosion damage for the iron and its alloys even if a trace amount of oxygen is present. On the other hand, almost all of the regular corrosion inhibitors do not work well when O_2_ is present [13]. Therefore, to comprehensively understand the corrosion mechanism and to predict the corrosion of steel in the oxygen-containing aqueous solution are highly significant. Although there are some studies which are related to corrosion mechanisms of oxygen corrosion [14,15,16,17,18,19,20,21,22], a further study on the corrosion mechanism and prediction of the iron alloys in oxygen-containing aqueous solution is still needed.

A few works about O_2_ corrosion of steel have been reported in the past few years, and most of them mainly focused on the effect of oxygen concentration on corrosion [14,15,16,17,18]. For example, Baek et al. [15] held the view that dissolved oxygen played a critical role in the formation of anodic oxide film and its growth kinetics on low carbon steel. A feature in common among these studies is that the effects of oxygen reduction, hydrogen reduction, water reduction and iron oxidation on corrosion are not quantitatively discussed.

In oxygen-containing aqueous solution, porous and non-protective oxides and oxyhydroxides such as goethite, lepidocrocite and magnetite [19] usually form on the steel surface, suggesting that the corrosion products do not affect the corrosion rate from the initial corrosion stage to the end of corrosion. Therefore, it can be considered that the corrosion behaviors and corrosion prediction at the initial stage is of high relevance to the whole corrosion process. Until now, a comprehensive study on the oxygen corrosion and corrosion prediction of steel at the initial stage is still missing. Cáceres et al. [20] developed a superposition model, in which the kinetic parameters about iron oxidation, hydrogen evolution and oxygen reduction were used to illustrate the relationship between corrosion rate and oxygen concentration or NaCl concentration. Unfortunately, the water reduction was not taken into account in this model. Actually, water reduction plays an important role in the corrosion in a solution with a high pH and a low oxygen concentration. Krawiec et al. [21,22] used a finite element approach to analyze electrochemical behaviors of 316 stainless steel in oxygen-containing solution, however, the agreement between the calculated value and experimental data was not good enough.

Low alloy steels are usually employed to manufacture the pressure vessel in the ground system in which the solution is often under static condition. In this work, the corrosion behaviors of Q345R steel in oxygen-containing solution at the initial stage will be investigated using electrochemical techniques. Then a numerical model based on the mixed potential theory will be proposed. The effects of oxygen concentration, temperature and pH on corrosion behaviors will be discussed.

## 2. Materials and Methods

### 2.1. Specimens and Solution

The specimens used in this work were cut from a Q345R steel plate with the following chemical composition (wt.%): C 0.203, Si 0.218, Al 0.275, Mn 1.324, P 0.031, S 0.041, Ni 0.045, Cr 0.072, V 0.054, Mo 0.061, with Fe making up the balance. The specimens were cylindrical, and were sealed in epoxy, with an exposed cross-sectional surface area of 1 cm^2^. Before the experiment, the electrode surface (exposed surface) was grounded sequentially using up to 800 grit SiC paper, degreased in an ultrasonic bath with acetone for 10 min, rinsed in ethanol, and dried in nitrogen, then immediately used for test.

**Figure 1:**
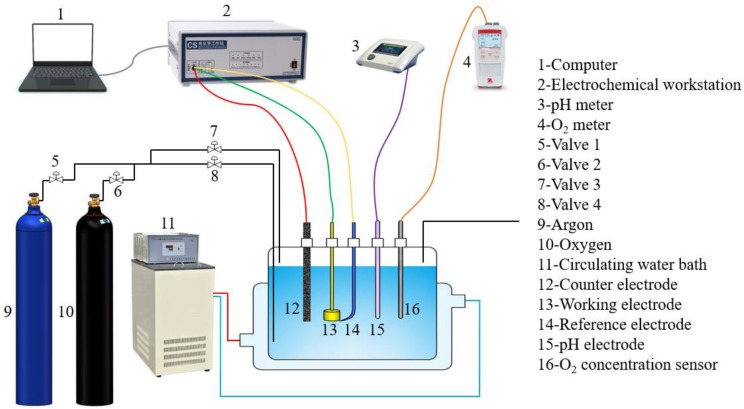
Schematic diagram of experimental setup.

The test solution was 1 wt.% NaCl, which was prepared from analytical grade regent and deionized water (18.25 MΩ·cm in resistivity). The dissolved oxygen in the solution was adjusted by purging the argon, or the mixture gas of argon and oxygen. Prior to each experiment, the argon or the mixture gas with different volume ratios of argon and oxygen was initially purged into the solution for 24 h to achieve a designated value of dissolved oxygen concentration. During the experiment, the gas purging was maintained to ensure a stable dissolved oxygen concentration. The content of oxygen was real time monitored using a STARTER 400D system (Ohaus Crop., Parsippany, NJ, USA) which had a detection accuracy of 0.01 mg/L (Figure 1). After the designated temperature was achieved, the solution pH was adjusted by deoxygenated dilute HCl or NaOH solution.

### 2.2. Electrochemical Measurements

The electrochemical measurements including electrochemical impedance spectroscopy (EIS), cathodic polarization curves and anodic polarization curves were carried out using a CS 350 electrochemical workstation (Wuhan Corrtest Instruments Corp. Ltd., Wuhan, China). All the potentials were with respect to saturated calomel electrode (SCE). A classical three-electrode setup was used, the prepared specimen was used as the working electrode, a platinum plate was used as the counter electrode, and a SCE was used as the reference electrode (Figure 1). To simulate the corrosion environment, a static solution system was employed in this work. In order to calculate the IR-drop between the working electrode and the reference electrode, EIS was used to measure the solution resistance. EIS measurements were performed at OCP with an oscillating potential ±5 mV in the frequency range from 100 kHz to 10 mHz. The cathodic polarization curve measurements were performed by shifting the potential from open circuit potential (OCP) to −500 mV vs. OCP with a scanning rate of 0.5 mV/s. After the measurement of the cathodic polarization curve, the electrode was then permitted to equilibrate back to the OCP. Afterwards, the anodic polarization curve of the same specimen was measured by shifting the potential from OCP to +150 mV vs. OCP with scanning rate of 0.5 mV/s. IR-drop in all the potential shown in this work has been manually deduced. To check the reproducibility, all the electrochemical measurements were repeated at least three times.

## 3. Modeling Description

The electrochemical model described in this work is based on the mixed potential theory. The following assumptions are made to establish the transient kinetic-transport equations applicable to a static state: (i) the homogeneous reactions are assumed, and the current distribution is uniform over the working electrode surface. Only transport from the bulk solution to the electrode surface is considered; (ii) the transport and kinetic parameters are uniform all over the system; (iii) ideally dilute behavior is presumed in the solution, i.e., activity coefficients are equal to 1; (iv) the donation of electro-migration and convection to mass transfer process are negligible because the solution contains an abundance of supporting electrolyte in absence of extra stir.

### 3.1. Cathodic Reactions

The reduction of oxygen, the reduction of H^+^ and the reduction of H_2_O in the cathodic reactions will be described, respectively.

#### 3.1.1. Reduction of Oxygen

The reduction of oxygen can be under the control of charge transfer and/or mass transfer (diffusion) [23]. Considering the mixed mass transfer and charge transfer control of the cathodic reaction rate, the effect of mass transfer on net current density $j_{\mathrm{net}}$ can be theoretically derived by Equation (1) (All the explanation about parameters, variables and constants in the modeling are summarized in Table 1, the same hereinafter):

(1) $$\frac{1}{j_{\mathrm{net}}}=\frac{1}{j_{\mathrm{ct}}}+\frac{1}{j_{\lim}}$$

where $j_{\mathrm{ct}}$ is the current density controlled by charge transfer and $j_{\lim}$ is the limiting current density controlled by mass transfer. The equation has been widely used in elementary mechanistic models of corrosion [23,24,25].

Consequently, the rate of oxygen reduction in terms of current density can be written:

(2) $$\frac{1}{j_{O_{2}}}=\frac{1}{j_{\mathrm{ct},O_{2}}}+\frac{1}{j_{\lim,O_{2}}}$$

The charge transfer current density $j_{\mathrm{ct},O_{2}}$ is calculated as:

(3) $$j_{\mathrm{ct},O_{2}}{=j}_{0,O_{2}}\times 10^{\frac{-\left(E-E_{\mathrm{rev},O_{2}}\right)}{b_{O_{2}}}}$$

The exchange current density $j_{0,O_{2}}$ is a function of pH and temperature [30]:

(4) $$j_{0,O_{2}}=j_{0,O_{2}}^{\mathrm{ref}}{\left(\frac{C_{H^{+}}^{b}}{C_{H^{+}}^{\mathrm{ref}}}\right)}^{-a_{O_{2}}}e^{\frac{-\Delta H_{O_{2}}}{R}\left(\frac{1}{T}-\frac{1}{T_{O_{2}}^{\mathrm{ref}}}\right)}$$

Without regard to polarization potential, the enthalpy of activation ΔH can replace the activation energy ΔE that explains the temperature dependence in the usual Arrhenius form [31]. The symmetry factor for O_2_ reduction which is temperature dependent in the range of 20–250 °C can be expressed as [27]:

(5) $$a_{O_{2}}=0.001678T$$

Due to the potential applied in corrosion process, the absolute value for cathodic Tafel slope $b_{O_{2}}$ used in this model is 120 mV/decade [29].

The reversible potential for O_2_ reduction is a function of temperature and pH:

(6) $$E_{\mathrm{rev},O_{2}}=E_{\mathrm{rev},O_{2}}^{0}+\frac{2.303RT}{4F}\log P_{O_{2}}-\frac{2.303RT}{F}\left(\mathrm{pH}-pK_{w}\right)$$

where the partial pressure of oxygen $P_{O_{2}}$ is normally set to 1 atm.

The dissociation constant of water with respect to temperature follows [32]:

(7) $$pK_{w}=29.3868-0.0737549T+7.747881\times 10^{-5}T^{2}$$

The limiting mass transfer current density $j_{\lim,O_{2}}$ is related to the rate of transport by oxygen diffusion from the bulk solution through the boundary layer to the steel surface [33]:

(8) $$j_{\lim,O_{2}}=\frac{4FD_{O_{2}}C_{O_{2}}^{b}}{\delta }$$

In a dilute solution, the relationship between diffusion coefficient of oxygen and temperature is based on Stokes-Einstein equation [28]:

(9) $$D_{O_{2}}=D_{\mathrm{ref},O_{2}}\times \frac{T}{T_{\mu }^{\mathrm{ref}}}\times \frac{\mu_{\mathrm{ref}}}{\mu }$$

The temperature dependence of water kinetic viscosity is given by [28]:

(10) $$\mu =\mu_{\mathrm{ref}}\times \;10^{\frac{1.3272\left(T_{\mu }^{\mathrm{ref}}-T\right)-0.001053{\left(T_{\mu }^{\mathrm{ref}}-T\right)}^{2}}{T-168.15}}$$

No matter how many species are involved in an equilibrium, only one diffusion boundary layer thickness δ must be considered [34]. According to the open literature [35,36], the diffusion boundary layer thickness is 0.4 mm at 30 °C, 0.35 mm at 60 °C and 0.21 mm at 90 °C. Apparently, the diffusion boundary layer thickness depends upon the temperature. However, the function between diffusion boundary layer thickness and temperature is still unknown. In this work, a quadratic equation involving temperature and diffusion boundary layer thickness is fitted from the opening values [35,36] of diffusion boundary layer thickness at different temperatures:

(11) $$\delta =-5\;\times 10^{-5}T^{2}+3.015\times 10^{-2}T-4.144$$

#### 3.1.2. Reduction of H^+^

Like the reduction of oxygen, the current density of H^+^ reduction can be described as:

(12) $$\frac{1}{j_{H^{+}}}=\frac{1}{j_{\mathrm{ct},H^{+}}}+\frac{1}{j_{\lim,H^{+}}}$$

The charge transfer current density $j_{\mathrm{ct},H^{+}}$ follows:

(13) $$j_{\mathrm{ct},H^{+}}{=j}_{0,H^{+}}\times 10^{\frac{-\left(E-E_{\mathrm{rev},H^{+}}\right)}{b_{H^{+}}}}$$

The exchange current density $j_{0,H^{+}}$ depends on pH and temperature [30]:

(14) $$j_{0,H^{+}}=j_{0,H^{+}}^{\mathrm{ref}}{\left(\frac{C_{H^{+}}^{b}}{C_{H^{+}}^{\mathrm{ref}}}\right)}^{0.5}e^{\frac{-\Delta H_{H^{+}}}{R}\left(\frac{1}{T}-\frac{1}{T_{H^{+}}^{\mathrm{ref}}}\right)}$$

usually, symmetry factor for H^+^ reduction $b_{H^{+}}$ seems to be independent of temperature and well-recognized value of 0.5 is widely employed [27]. Therefore, the absolute value of cathodic Tafel slope can be expressed as follows:

(15) $$b_{H^{+}}=\frac{2.303RT}{0.5F}$$

The reversible potential for H^+^ reduction $E_{\mathrm{rev},H^{+}}$ is calculated as:

(16) $$E_{\mathrm{rev},H^{+}}=E_{\mathrm{rev},H^{+}}^{0}-\frac{2.303RT}{2F}\log P_{H_{2}}-\frac{2.303RT}{F}\mathrm{pH}$$

where the partial pressure of hydrogen $P_{H_{2}}$ is normally set to 1 atm.

The current density of limiting diffusion of H^+^ could be considered as:

(17) $$j_{\lim,H^{+}}=\frac{FD_{H^{+}}C_{H^{+}}^{b}}{\delta }$$

Similar to diffusion coefficient of O_2_, the diffusion coefficient of H^+^ can be calculated using Equation (18):

(18) $$D_{H^{+}}=D_{\mathrm{ref},H^{+}}\times \frac{T}{T_{\mu }^{\mathrm{ref}}}\times \frac{\mu_{\mathrm{ref}}}{\mu }$$

#### 3.1.3. Reduction of Water

In the aqueous solution, the number of water molecules on the electrode interface can be seen as unlimited. Therefore, the reduction rate of water is under charge transfer control. Hence, using pure Tafel equation as:

(19) $$j_{H_{2}O}{=j}_{0,H_{2}O}\times 10^{\frac{-\left(E-E_{\mathrm{rev},H_{2}O}\right)}{b_{H_{2}O}}}$$

The exchange current density $j_{0,H_{2}O}$ can be described as Equation (20) [30]:

(20) $$j_{0,H_{2}O}=j_{0,H_{2}O}^{\mathrm{ref}}{\left(\frac{C_{H^{+}}^{b}}{C_{H^{+}}^{\mathrm{ref}}}\right)}^{-0.5}e^{\frac{-\Delta H_{H_{2}O}}{R}\left(\frac{1}{T}-\frac{1}{T_{H_{2}O}^{\mathrm{ref}}}\right)}$$

The absolute value of cathodic Tafel slope of water reduction $b_{H_{2}O}$ can be expressed as Equation (21):

(21) $$b_{H_{2}O}=\frac{2.303RT}{0.5F}$$

The reversible potential $E_{\mathrm{rev},H_{2}O}$ is respected to:

(22) $$E_{\mathrm{rev},H_{2}O}=E_{\mathrm{rev},H_{2}O}^{0}-\frac{2.303RT}{2F}\log P_{H_{2}}-\frac{2.303RT}{F}\left(\mathrm{pH}-pK_{w}\right)$$

The reversible potential of water is the same as that of H^+^. The partial pressure of hydrogen normally is set to 1 atm.

### 3.2. Anodic Reaction

Although steel contains a series of elements, the electrochemical dissolution of iron in an aqueous solution is the main anodic reaction [37]. Thus, only the oxidation of iron is taken into account in anodic reaction in this work. This reaction is controlled by charge transfer process and it is independent of oxygen concentration. Hence, pure Tafel behavior can be assumed close to the corrosion potential, as described in Equation (23).

(23) $$j_{\mathrm{Fe}}=j_{0,\mathrm{Fe}}\times 10^{\frac{E-E_{\mathrm{rev},\mathrm{Fe}}}{b_{\mathrm{Fe}}}}$$

The standard electrode potential of iron (−0.447 V vs. SHE) is used to represent $E_{\mathrm{rev},\mathrm{Fe}}$ in Equation (23) [26]. Nesic et al. [38] suggested that the concentration of ferrous ions in solution does not affect the dissolution kinetics of iron in the absence of film formation. Even if the overall anodic reaction does not suggest any dependence on pH, numerous studies have revealed that in strong acidic solutions the reaction order with respect to OH^−^ is between 1 and 2 [37,38,39]. For example, Nesic et al. [38] illuminated that the exchange current density of iron in a solution without CO_2_:

(24) $$\frac{\partial \mathrm{lg}j_{0,\mathrm{Fe}}}{\partial \mathrm{pH}}=1$$

between pH 3 and 4, while it changed very little between pH 4 and 5, which was in agreement with findings of Bockris [37].

Then the exchange current density of iron oxidation is a function of temperature and H^+^ concentration [30,37,38]:

(25) $$j_{0,\mathrm{Fe}}=j_{0,\mathrm{Fe}}^{\mathrm{ref}}{\left(\frac{C_{H^{+}}^{b}}{C_{H^{+}}^{\mathrm{ref}}}\right)}^{a_{1}}e^{\frac{-\Delta H_{\mathrm{Fe}}}{R}\left(\frac{1}{T}-\frac{1}{T_{\mathrm{Fe}}^{\mathrm{ref}}}\right)}$$

it should be noted that $a_{1}=-1$ if pH < 4, otherwise, $a_{1}=0$. In the opening literature, the measured Tafel slopes typically range from 30 to 80 mV/decade at different conditions [36]. In this model, 70 mV/decade is used.

### 3.3. The Mixed Potential Theory

The corrosion potential $E_{\mathrm{corr}}$ can be calculated from the charge balance equation at the steel surface:

(26) $$j_{\mathrm{corr}}{=j}_{\mathrm{Fe}}{=j}_{O_{2}}{+j}_{H^{+}}{+j}_{H_{2}O}$$

It expresses the simple fact that at stable state all the electrons consumed by the sum of the cathodic processes is generated by anodic process. Once the corrosion potential is calculated, the corrosion current density $j_{\mathrm{corr}}$ and corrosion rate CR can be calculated from anodic current density or total cathodic current density at the corrosion potential:

(27) $$CR=0.1634\frac{j_{\mathrm{corr}}M}{\rho }$$

## 4. Results and Discussion

### 4.1. The Characteristics of the Polarization Curves and Modeling Curves

**Figure 2:**
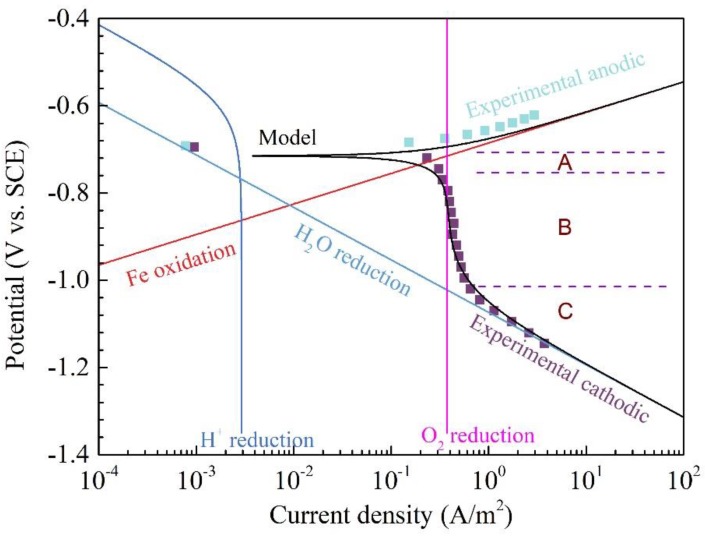
The polarization curves and its modeling results of Q345R steel in 1 wt.% NaCl solution containing 4.16 mg/L oxygen, with pH 6.0 at 30 °C. Square: experimental data. Solid line: modeling result.

Figure 2 shows the polarization curves of Q345R steel in 1 wt.% NaCl solution containing 4.16 mg/L oxygen with pH 6.0 at 30 °C. It can be seen that the cathodic polarization curve could be divided into three regions with the negative shift of potential. Since the solution pH is equal to 6.0, the concentration of H^+^ is relatively low. Therefore, region A should be mainly associated with the reduction of dissolved oxygen at the electrode/solution interface, as given by (28).

(28) $$O_{2}+2H_{2}O+4e^{-}\to 4OH^{-}$$

Region B is the diffusion-limited current density region, which is mainly controlled by the diffusion of oxygen. When the cathodic polarization potential moves to region C, the reduction of H_2_O becomes the main reaction on the electrode surface, as given by (29):

(29) $$2H_{2}O+2e^{-}\to H_{2}+2OH^{-}$$

For the anodic polarization curve, it is observed that the current density increases sharply with the positive shift of potential. Although Q345R steel is composed of several elements, iron is the most abundant element. Therefore, the oxidation of iron in an aqueous solution should be the main anodic reaction given by (30).

(30) $$\mathrm{Fe}\to Fe^{2+}+2e^{-}$$

As a comparision, the current density vs. potential curves of the H^+^ reduction, oxygen reduction, water reduction, Fe oxidation and the total polarization curves calculated from the model are also shown in Figure 2. Generally, the total polarization curves calculated from the model show a good agreement with the corresponding experimental data. Therefore, the corrosion current density or the corrosion rate of Q345R steel in the test condition can be easily calculated according to this model. Moreover, the experimental data can also be quantitatively analyzed using this model. For example, the contribution of H^+^ reduction to the total current density at corrosion potential is very small (below 2.91 × 10^−3^ A/m^2^). This is why the oxygen reduction in the regions A and B is the main cathodic reaction, rather than the H^+^ reduction. However, as soon as the current density of oxygen reduction increases up to the value of diffusion-limited current density, it does not increase any more even if the potential further shifts negatively. The current density of water reduction would be bigger than that of oxygen reduction when the potential shift below −1.01 V vs. SCE. Therefore, in the region C, the water reduction becomes the main cathodic reaction.

**Figure 3:**
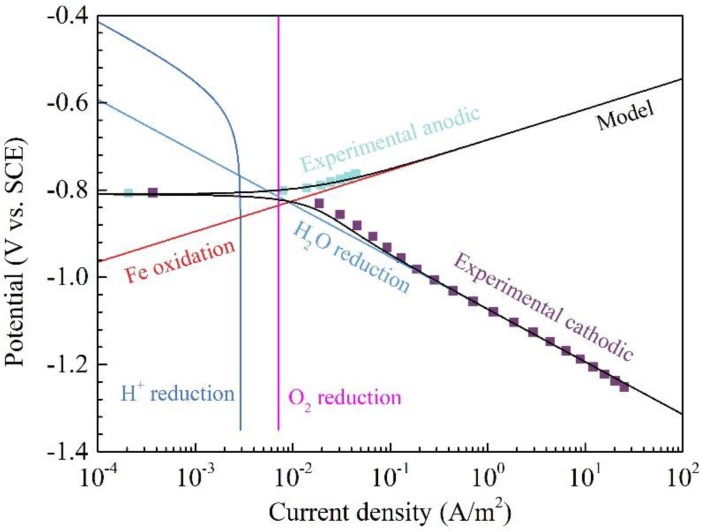
The polarization curves and modeling results of Q345R steel in 1 wt.% NaCl solution containing 0.08 mg/L oxygen, with pH 6.0 at 30 °C. Square: experimental data. Solid line: modeling result.

To further understand the characteristics of the polarization curves, the polarization curves and the corresponding modeling curves of Q345R steel in 1 wt.% NaCl solution containing 0.08 mg/L oxygen with pH 6.0 at 30 °C are also shown in Figure 3. Apparently, the total modeling results also show a good agreement with the experimental data. The current density of oxygen reduction (about 7.19 × 10^−3^ A/m^2^) is so low that the oxygen reduction characteristics almost disappear on the cathodic polarization curves regardless of the experimental data or the modeling results. This means that the water reduction should almost always be the main cathodic reaction. The modeling curve of water reduction shown in Figure 3 almost overlaps with the cathodic polarization curve, this also demonstrates that the water reduction is the main cathodic reaction. In addition, the current density of H^+^ reduction does not change when the oxygen concentration decreases from 4.16 mg/L to 0.08 mg/L, because the pH in both solutions is identical.

According to the discussed above, it can be concluded that the model proposed in this work can be used to quantitatively analyze the corrosion behaviors or to predict the corrosion rate of Q345R steel at the initial corrosion stage in oxygen-containing aqueous solution. The effects of oxygen concentration, temperature and pH on the corrosion behaviors will be discussed in detail in the following sections using combined experimental data and modeling results.

### 4.2. Effect of Oxygen Concentration on the Corrosion of Q345R Steel

**Figure 4:**
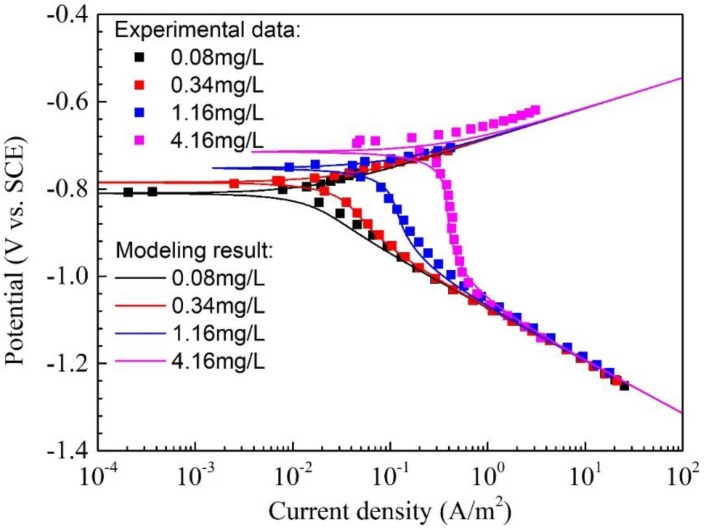
The polarization curves and modeling results of Q345R steel in 1 wt.% NaCl solution with different oxygen concentrations at pH 6.0 and 30 °C. Square: experimental data. Solid line: modeling result.

Figure 4 shows the polarization curves and modeling curves of Q345R steel in 1 wt.% NaCl solution with different oxygen concentrations at pH 6.0 and 30 °C. The corrosion potential, corrosion current density and the corrosion rate calculated from the experimental data or model are listed in Table 2. It can be found that the oxygen concentration greatly affects the corrosion behaviors of Q345R steel in the test solution. According to the experimental results, the corrosion potential increases with the increasing oxygen concentration. E.g., as the oxygen concentration increases from 0.08 mg/L to 4.16 mg/L, the corrosion potential shifts positively from −0.807 V vs. SCE to −0.694 V vs. SCE. This indicates that the corrosion of Q345R steel in the test solution is determined by the cathodic reaction. At the same time, the corrosion current density changes from 0.017 A/m^2^ to 0.377 A/m^2^ and rises over 22 times when the oxygen concentration increases from 0.08 mg/L to 4.16 mg/L. This can be attributed to that the cathodic reaction is accelerated by oxygen with higher concentration.

No passivity feature is observed, and the steel is in an active dissolution state. The characteristics of the anodic polarization curves do not change significantly with the increasing oxygen concentration, however, great changes in cathodic polarization curves can be found. The high oxygen concentration, the higher reduction density is, hence the higher corrosion rate. When the oxygen concentration increases from 0.08 mg/L to 4.16 mg/L, the current density of oxygen reduction at the corrosion potential rises from 0.007 A/m^2^ to 0.374 A/m^2^, and the contribution of oxygen reduction current density to the corrosion current density increases from 43.11% to 99.12%. The plateau formed in the limiting diffusion region gradually becomes clear when the oxygen concentration increases from 0.08 mg/L to 4.16 mg/L. E.g., for oxygen concentrations of 0.08 mg/L and 0.34 mg/L, the plateau of limiting diffusion can hardly be seen. The curve for each oxygen concentration is almost a straight line in the strong polarization region and its slope is close to the slope of the water reduction reaction, demonstrating that the water reduction reaction is the main cathodic reaction (See Figure 4). As the oxygen concentration increases to 1.16 mg/L, this plateau is present. When the oxygen further increases up to 4.16 mg/L, a typical plateau for the limiting diffusion of oxygen has been revealed on the curve.

According to the comparison of the experimental data with the modeling curves, it can be found that the agreement between both for all the oxygen concentration conditions is good, as it is shown in Figure 4. The corrosion potential and corrosion current density calculated from the model are also close to the corresponding values fitted from the experiment data (Table 2). E.g., the maximum deviation is 0.012 A/m^2^ and the variance is only 3.63 × 10^−5^ for the corrosion current density. These results demonstrate that the proposed model can be used to predict the corrosion behaviors and corrosion rate of Q345R steel in the aqueous solutions with different oxygen concentrations at pH 6.0.

### 4.3. Effect of Temperature on the Corrosion of Q345R Steel

**Figure 5:**
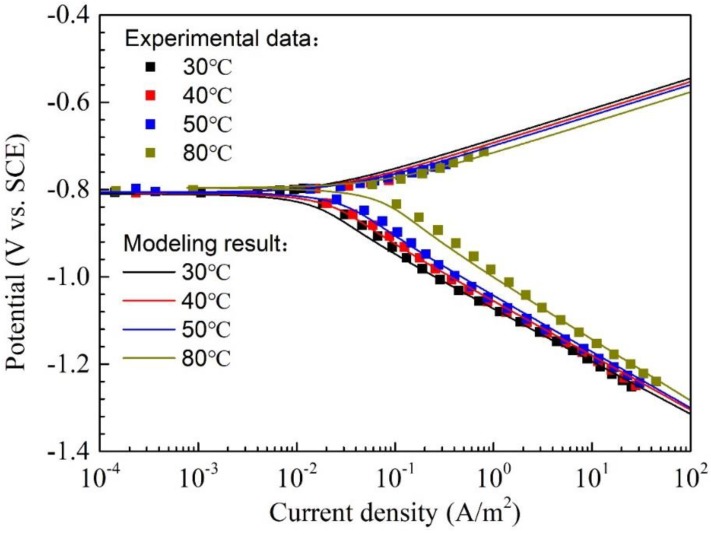
The polarization curves and modeling results of Q345R steel in 1 wt.% NaCl solution with 0.08 mg/L oxygen at pH 6.0 under different temperatures. Square: experimental data. Solid line: modeling result.

The environmental temperature may be different when the lines and equipment is under various working conditions. Therefore, it is necessary to investigate the effect of temperature on the corrosion behaviors of Q345R steel. Figure 5 shows polarization curves and modeling results of Q345R steel in 1 wt.% NaCl solution with 0.08 mg/L oxygen at pH 6.0 under different temperatures. It is seen that there is no plateau for limiting diffusion on the curves obtained from all temperatures, this is due to the low oxygen concentration (0.08 mg/L) in the test solution, as discussed above. The slope of each cathodic polarization curve at strong polarization region changes little and its value is very close to the slope of water reduction reaction. According to the discussion in the Section 4.2, it is easy to know that the corrosion process is controlled by the water reduction reaction under all the temperatures. The corrosion potential, corrosion current density and the corrosion rate fitted from the experimental data or calculated from the model are summarized in Table 3. The change in corrosion potential is just a few millivolts as the temperature increases from 30 °C to 80 °C. However, there is a continuous and obvious increase in corrosion current density and corrosion rate as the temperature increases. E.g., the corrosion rate at 80 °C is 0.088 mm/y which is about 4.6 times than the corrosion rate at 30 °C. Temperature plays a significant role in the whole corrosion process, accelerating electrochemical, chemical, mass transfer, etc. Therefore, one could expect that the corrosion rate steadily increases with temperature when precipitation of protective layers does not occur. At last, the agreement between the experimental data and modeling curves is almost perfect, the variance of corrosion current density is only 1.50 × 10^−6^, indicating that the proposed model is valid at pH 6.0 under the temperature from 30 °C to 80 °C.

### 4.4. Effect of pH on the Corrosion of Q345R Steel

**Figure 6:**
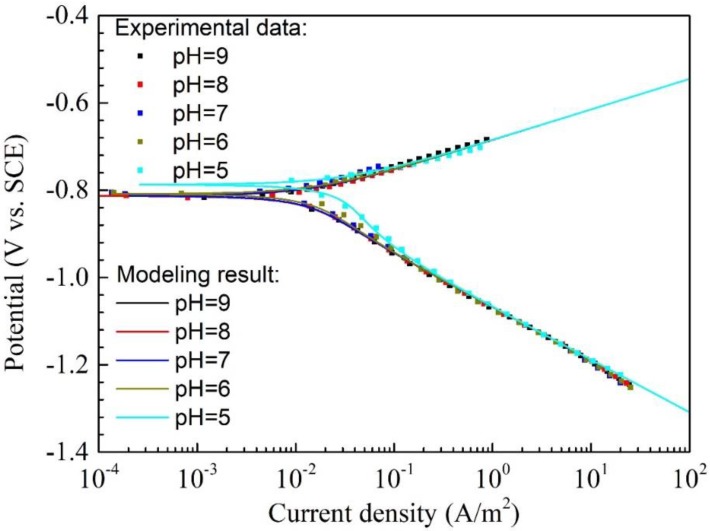
The polarization curves and modeling results of Q345R steel in 1 wt.% NaCl solution with 0.08 mg/L oxygen at different pH under 30 °C. Square: experimental data. Solid line: modeling result.

Figure 6 shows the polarization curves and the modeling results of Q345R steel in 1 wt.% NaCl solution containing 0.08 mg/L oxygen with pH 9.0 to 5.0 at 30 °C. Generally, it is shown that the modeling results are in good agreement with the experimental data. The parameters including corrosion potential, corrosion current density and corrosion rate calculated from the model are also very close to the corresponding values fitted from the experimental data (See Table 4). The variance of corrosion current density is only 4.20 × 10^−6^. This demonstrates that the proposed model is valid in the pH range of 9.0 to 5.0.

It is interesting that the curves are almost overlapped together from pH 9.0 to 6.0, and the corrosion potential as well as corrosion current density change little in this pH range. This means that the corrosion behavior is nearly independent of pH in this range. However, as the pH decreases down to 5.0, the corrosion potential rises obviously and the corrosion current density also increases remarkably. This can be ascribed to that the current density of H^+^ reduction at pH 5.0 is much higher than other pH in the range of 9.0 to 6.0. E.g., the H^+^ reduction current density for the pH 5.0 is 2.309 × 10^−2^ A/m^2^ at −0.82 V vs. SCE, however, the current density of H^+^ reduction for pH 9.0 and 6.0 at the same potential is only 2.308 × 10^−6^ A/m^2^ and 2.309 × 10^−3^ A/m^2^, respectively. In addition, the contribution of H^+^ reduction current density at −0.82 V vs. SCE increases from 0.01% to 45.25% as pH decreases from 9.0 to 5.0.

**Figure 7:**
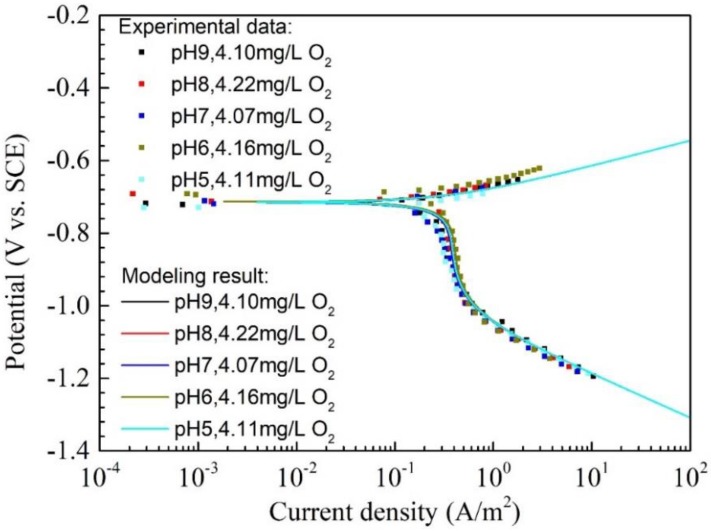
The polarization curves and modeling results of Q345R steel in 1 wt.% NaCl solution containing 4.07 to 4.22 mg/L oxygen at different pH under 30 °C. Square: experimental data. Solid line: modeling result.

Figure 7 shows polarization curves and modeling results of Q345R steel in 1 wt.% NaCl solutions containing high oxygen concentrations (from 4.07 mg/L to 4.22 mg/L) with pH 9.0 to 5.0 at 30 °C. It should be pointed out that it is very hard to control the oxygen concentration in each solution at a completely identical value for all the experiments because of the limitation in the accuracy of gas flow meter. Therefore, oxygen concentrations around 4.16 mg/L are used during the experiments in this work. As is shown in Figure 7, there are no big differences among the curves for all the pH from 9.0 to 5.0 and all the curves are overlapped together. This phenomenon can also be confirmed by the corrosion potential and corrosion current density obtained from either experimental data or model, as listed in Table 5. This demonstrates that the corrosion behavior of Q345R steel in solution with around 4.16 mg/L oxygen is almost independent of pH in the range of 9.0 to 5.0, i.e., the dedication of H^+^ reduction to corrosion is relatively small. In contrast to the corrosion behavior for the 0.08 mg/L oxygen shown in Figure 6, the corrosion behavior in the presence of around 4.16 mg/L oxygen for the pH 5.0 does not show any difference from other pH in the range of 9.0 to 6.0. This can be attributed to that the oxygen reduction is the main cathodic reaction in the potential range where the water reduction has not yet started to control the whole cathodic reaction. In this potential range, the current density of H^+^ reduction is much smaller than oxygen reduction. E.g., when the oxygen concentration is 4.11 mg/L, the O_2_ reduction current density at the corrosion potential for the pH 5.0 is 0.369 A/m^2^, which is 16.89 times higher than the current density of H^+^ reduction. Furthermore, the O_2_ reduction current density at the corrosion potential for pH 9.0, 4.10 mg/L O_2_ is up to 1.69 × 10^5^ times higher than the current density of H^+^ reduction.

**Figure 8:**
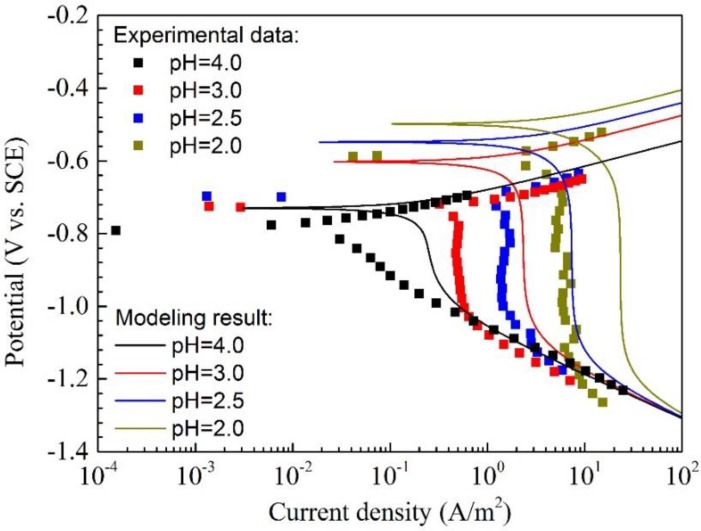
The polarization curves and modeling results of Q345R steel in 1 wt.% NaCl solution containing 0.08 mg/L oxygen with different low pH at 30 °C. Square: experimental data. Solid line: modeling result.

As shown in Figure 6, the corrosion behavior of Q345R steel in the 1 wt.% NaCl solution containing 0.08 mg/L oxygen at pH 5.0 is remarkably different from that of the higher pH (from 9.0 to 6.0) due to the presence of stronger H^+^ reduction. So, what will happen in the solution with lower pH? In this context, the polarization curves of Q345R steel in 1 wt.% NaCl solution containing 0.08 mg/L oxygen at pH from 4.0 to 2.0 are measured, as is shown in Figure 8. It can be seen that the plateau of limiting diffusion of H^+^ on each curve is present for pH 3.0, 2.5 and 2.0, which is completely different from the curves measured at higher pH (e.g., pH 9.0 to 5.0) with the same oxygen concentration under the same temperature. Moreover, the data on the cathodic polarization curves is scattering (See Figure 8), indicating that there are disturbances during the measurements.

The modeling curves are also plotted in the Figure 8 for comparison. Unfortunately, it can be found that the modeling curves, especially for the cathodic polarization curves, completely disaccords with the corresponding experimental data. In general, the current density calculated from the model is much higher than that fitted from the experimental data. E.g., the current density at −0.9 V vs. SCE calculated from the model at pH 3.0 is 2.324 A/m^2^ which is 6.25 times the fitted result from the experimental data. As mentioned above, some disturbances are present during the measurements of cathodic polarization curves, resulting in the appearance of scattering data on the cathodic polarization curves. This disturbance most probably comes from the dramatic reaction at the electrode/solution interface, since the whole solution is under static condition without any special disturbance from outside.

**Figure 9:**
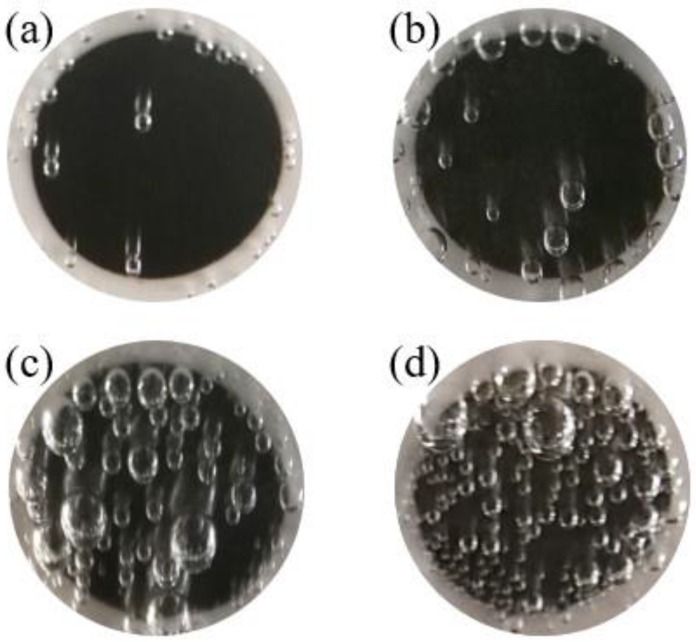
Optical photograph of Q345R steel in 1wt.% NaCl solution at 30 °C, 0.08 mg/L oxygen, −0.9V vs. SCE: (**a**) pH 4.0; (**b**) pH 3.0; (**c**) pH 2.5; and (**d**) pH 2.0.

After taking all the reactions occurred at the electrode/solution interface into consideration, the hydrogen evolution should be the only reaction which could make the experimental data scattered on the polarization curves. Figure 9 shows the optical photos of Q345R steel electrode in 1wt.% NaCl solution containing 0.08 mg/L oxygen at different pH under −0.9 V vs. SCE. It is clear to see that the number of hydrogen bubbles increases with the decreasing pH at the same level of cathodic polarization. Besides, it is also possible that some invisible hydrogen bubble formed on the electrode surface. On the one hand, the collapse of the gas bubbles can generate the disturbances on the electrode/solution interface, resulting in the presence of scattering data on the cathodic polarization curves. On the other hand, the actual area of reaction on the electrode surface is greatly reduced by the adsorbed gas bubbles which can partially block the contact between solution and electrode surfaces. The similar phenomenon can also be found in the literature [40,41]. Consequently, the current density fitted from the experimental data is much lower than those calculated from the model, because a preset electrode area which is much larger than the actual one is used during the measurement. This can successfully explain why the modeling curve is not in agreement with the experimental data. However, the quantitative variation in electrode area during the cathodic polarization in low pH is still unknown. This is very important to the modeling and prediction of corrosion under static condition in acidic media. Besides, there are also some other possible reasons for discrepancy between experimental data and modelling results. Firstly, H^+^ concentration near to electrode surface would be not completely equal to zero, but it would stabilize at a low value, especially when pH at a low value in the actual condition. This could cause the modelling results deviate from the experimental results. Secondly, there is lack of evidence to describe that whether the diffusion boundary layer thickness would increase or not with the decrease in pH. Unfortunately, quantitative analysis of these mentioned effects on corrosion is rather difficult. The related work is still in progress and it will be shown in our future work.

## 5. Conclusions

According to the experimental results, the corrosion rate of Q345R steel at the initial stage in 1 wt.% NaCl solution increases with the increasing oxygen concentration from 0.08 mg/L to around 4.16 mg/L. The higher temperature, the higher the corrosion rate. Corrosion behaviors change little as the pH decreases from 9.0 to 5.0. However, lower pH greatly affects the corrosion behaviors.

The proposed model shows a good agreement with the experimental data in the pH range from 9.0 to 5.0. In this pH range, the oxygen reduction reaction, H^+^ reduction, the water reduction, and the iron oxidation can be quantitatively analyzed using this model. However, this model shows a disagreement with the experimental data at lower pH. This can be attributed to the actual area of reaction on the electrode being much smaller than the preset area due to the block effect from hydrogen bubbles adsorbed on the electrode surface.

## Figures and Tables

**Table 1:** Model parameters, variables and constants.

Symbol	Value	Unit	Definition	Reference
Standard potential (vs. SHE)				
$E_{\mathrm{rev},\mathrm{Fe}}^{0}$	−0.447	V	$\mathrm{Fe}↔{\mathrm{Fe}}^{2+}{+2e}^{-}$	[26]
$E_{\mathrm{rev},O_{2}}^{0}$	0.401	V	$O_{2}+2H_{2}O+4e^{-}↔4OH^{-}$	[26]
$E_{\mathrm{rev},H^{+}}^{0}$	0	V	$2H^{+}+2e^{-}↔H_{2}$	[26]
$E_{\mathrm{rev},H_{2}O}^{0}$	−0.828	V	$2H_{2}O+2e^{-}↔H_{2}+2OH^{-}$	[26]
Reference exchange current density				
$j_{0,\mathrm{Fe}}^{\mathrm{ref}}$	1	A/m^2^	$\mathrm{Fe}↔{\mathrm{Fe}}^{2+}{+2e}^{-}$	[24]
$j_{0,O_{2}}^{\mathrm{ref}}$	2.8 × 10^−3^	A/m^2^	$O_{2}+2H_{2}O+4e^{-}↔4OH^{-}$	[27]
$j_{0,H^{+}}^{\mathrm{ref}}$	0.03	A/m^2^	$2H^{+}+2e^{-}↔H_{2}$	[24]
$j_{0,H_{2}O}^{\mathrm{ref}}$	1.4 × 10^−5^	A/m^2^	$2H_{2}O+2e^{-}↔H_{2}+2OH^{-}$	[24]
Active enthalpy for exchange current density				
$\Delta H_{\mathrm{Fe}}$	37,500	J/mol	Fe oxidation	[24]
$\Delta H_{O_{2}}$	23,200	J/mol	O_2_ reduction	[27]
$\Delta H_{H^{+}}$	30,000	J/mol	H^+^ reduction	[24]
$\Delta H_{H_{2}O}$	30,000	J/mol	H_2_O reduction	[24]
Reference temperature				
$T_{\mathrm{Fe}}^{\mathrm{ref}}$	298.15	K	Exchange current density of $\mathrm{Fe}↔{\mathrm{Fe}}^{2+}{+2e}^{-}$	[24]
$T_{O_{2}}^{\mathrm{ref}}$	303.15	K	Exchange current density of $O_{2}+2H_{2}O+4e^{-}↔4OH^{-}$	[27]
$T_{H^{+}}^{\mathrm{ref}}$	298.15	K	Exchange current density of $2H^{+}+2e^{-}↔H_{2}$	[24]
$T_{H_{2}O}^{\mathrm{ref}}$	293.15	K	Exchange current density of $2H_{2}O+2e^{-}↔H_{2}+2OH^{-}$	[24]
$T_{\mu }^{\mathrm{ref}}$	293.15	K	Water dynamic viscosity	[28]
Reference physical parameters				
$C_{H^{+}}^{\mathrm{ref}}$	0.1	mol/m^3^	H^+^ concentration for exchange current density	[24]
$\mu_{\mathrm{ref}}$	1.002	kg/m·s	Water dynamic viscosity at 293.15K	[26]
Reference diffusion coefficient at 298.15K				
$D_{\mathrm{ref},O_{2}}$	2.3 × 10^−9^	m^2^/s	O_2_	[26]
$D_{\mathrm{ref},H^{+}}$	9.31 × 10^−9^	m^2^/s	H^+^	[26]
Constants				
*F*	96,485	C/mol	Faraday’s constant	[26]
*R*	8.314	J/mol·K	Universal gas constant	[26]
*M*	56	g/mol	Molecular mass of Fe	[26]
*ρ*	7.8	g/cm^3^	Density of Fe	[26]
Net current density				
$j_{\mathrm{corr}}$		A/m^2^	Corrosion	
$j_{\mathrm{Fe}}$		A/m^2^	Fe oxidation	
$j_{O_{2}}$		A/m^2^	O_2_ reduction	
$j_{H^{+}}$		A/m^2^	H^+^ reduction	
$j_{H_{2}O}$		A/m^2^	H_2_O reduction	
Exchange current density				
$j_{0,\mathrm{Fe}}$		A/m^2^	$\mathrm{Fe}↔{\mathrm{Fe}}^{2+}{+2e}^{-}$	
$j_{0,O_{2}}$		A/m^2^	$O_{2}+2H_{2}O+4e^{-}↔4OH^{-}$	
$j_{0,H^{+}}$		A/m^2^	$2H^{+}+2e^{-}↔H_{2}$	
$j_{0,H_{2}O}$		A/m^2^	$2H_{2}O+2e^{-}↔H_{2}+2OH^{-}$	
Charge transfer current density				
$j_{\mathrm{ct},O_{2}}$		A/m^2^	O_2_ reduction	
$j_{\mathrm{ct},H^{+}}$		A/m^2^	H^+^ reduction	
Limited current density				
$j_{\lim,O_{2}}$		A/m^2^	O_2_ reduction	
$j_{\lim,H^{+}}$		A/m^2^	H^+^ reduction	
Potential (vs. SHE)				
E		V	Steel potential	
$E_{\mathrm{corr}}$		V	Corrosion	
$E_{\mathrm{rev},\mathrm{Fe}}$		V	Reversible potential for $\mathrm{Fe}↔{\mathrm{Fe}}^{2+}{+2e}^{-}$	
$E_{\mathrm{rev},O_{2}}$		V	Reversible potential for $O_{2}+2H_{2}O+4e^{-}↔4OH^{-}$	
$E_{\mathrm{rev},H^{+}}$		V	Reversible potential for $2H^{+}+2e^{-}↔H_{2}$	
$E_{\mathrm{rev},H_{2}O}$		V	Reversible potential for $2H_{2}O+2e^{-}↔H_{2}+2OH^{-}$	
Absolute value for Tafel slope				
$b_{\mathrm{Fe}}$	70	mV/decade	Fe oxidation	
$b_{O_{2}}$	120	mV/decade	O_2_ reduction	[29]
$b_{H^{+}}$		mV/decade	H^+^ reduction	
$b_{H_{2}O}$		mV/decade	H_2_O reduction	
Diffusion coefficient				
$D_{O_{2}}$		m^2^/s	O_2_	
$D_{H^{+}}$		m^2^/s	H^+^	
Concentration in bulk solution				
$C_{O_{2}}^{b}$		mol/m^3^	O_2_	
$C_{H^{+}}^{b}$		mol/m^3^	H^+^	
Physical parameters				
*μ*		kg/m·s	Water dynamic viscosity	
T		K	Experiment temperature	
*δ*		mm	Diffusion boundary layer thickness	
*K_w_*		mol^2^/L^2^	Ionic product of water	
*CR*		mm/y	Corrosion rate	

**Table 2:** The corrosion potential, corrosion current density and corrosion rate of Q345R steel in 1 wt.% NaCl solution with different oxygen concentrations at pH 6.0 and 30 °C, including the results fitted from the experiment data and calculated from the model.

$C_{O_{2}}$ (mg/L)	Experimental Data			Modeling Result		
	$E_{\mathrm{corr}}$ (V vs. SCE)	$j_{\mathrm{corr}}$ (A/m^2^)	CR (mm/y)	$E_{\mathrm{corr}}$ (V vs. SCE)	$j_{\mathrm{corr}}$ (A/m^2^)	CR (mm/y)
0.08	−0.807 ± 0.004	0.017 ± 0.002	0.019 ± 0.002	−0.810	0.017	0.019
0.34	−0.787 ± 0.002	0.038 ± 0.002	0.046 ± 0.002	−0.785	0.037	0.044
1.16	−0.752 ± 0.004	0.097 ± 0.004	0.113 ± 0.005	−0.752	0.109	0.128
4.16	−0.694 ± 0.002	0.377 ± 0.005	0.443 ± 0.006	−0.715	0.377	0.443

**Table 3:** The corrosion potential, corrosion current density and corrosion rate of Q345R steel in 1 wt.% NaCl solution with 0.08 mg/L oxygen at pH 6.0 under different temperatures, including the results fitted from the experiment data and calculated from the model.

T (°C)	Experimental Data			Modeling Result		
	$E_{\mathrm{corr}}$ (V vs. SCE)	$j_{\mathrm{corr}}$ (A/m^2^)	CR (mm/y)	$E_{\mathrm{corr}}$ (V vs. SCE)	$j_{\mathrm{corr}}$ (A/m^2^)	CR (mm/y)
30	−0.807 ± 0.004	0.017 ± 0.002	0.019 ± 0.002	−0.810	0.017	0.019
40	−0.802 ± 0.006	0.022 ± 0.002	0.026 ± 0.002	−0.808	0.023	0.027
50	−0.793 ± 0.006	0.030 ± 0.002	0.035 ± 0.002	−0.806	0.031	0.036
80	−0.801 ± 0.008	0.075 ± 0.004	0.088 ± 0.005	−0.796	0.073	0.086

**Table 4:** The corrosion potential, corrosion current density and corrosion rate of Q345R steel in 1 wt.% NaCl solution with 0.08 mg/L oxygen at different pH under 30 °C, including the results fitted from the experiment data and calculated from the model.

pH	Experimental Data			Modeling Result		
	$E_{\mathrm{corr}}$ (V vs. SCE)	$j_{\mathrm{corr}}$ (A/m^2^)	CR (mm/y)	$E_{\mathrm{corr}}$ (V vs. SCE)	$j_{\mathrm{corr}}$ (A/m^2^)	CR (mm/y)
9.0	−0.815 ± 0.003	0.010 ± 0.001	0.012 ± 0.001	−0.814	0.014	0.017
8.0	−0.812 ± 0.002	0.012 ± 0.001	0.014 ± 0.001	−0.814	0.014	0.017
7.0	−0.805 ± 0.006	0.015 ± 0.001	0.018 ± 0.001	−0.814	0.014	0.017
6.0	−0.807 ± 0.004	0.017 ± 0.002	0.019 ± 0.002	−0.810	0.017	0.019
5.0	−0.787 ± 0.004	0.040 ± 0.002	0.047 ± 0.002	−0.783	0.040	0.047

**Table 5:** The corrosion potential, corrosion current density and corrosion rate of Q345R steel in 1 wt.% NaCl solution 4.07 to 4.22 mg/L oxygen at different pH under 30 °C, including the results fitted from the experiment data and calculated from the model.

Condition	Experimental Data			Modeling Result		
	$E_{\mathrm{corr}}$ (V vs. SCE)	$j_{\mathrm{corr}}$ (A/m^2^)	CR (mm/y)	$E_{\mathrm{corr}}$ (V vs. SCE)	$j_{\mathrm{corr}}$ (A/m^2^)	CR (mm/y)
pH 9.0, 4.10 mg/L oxygen	−0.718 ± 0.004	0.353 ± 0.008	0.414 ± 0.010	−0.715	0.369	0.433
pH 8.0, 4.22 mg/L oxygen	−0.707 ± 0.008	0.376 ± 0.005	0.441 ± 0.006	−0.715	0.380	0.446
pH 7.0, 4.07 mg/L oxygen	−0.717 ± 0.004	0.323 ± 0.009	0.379 ± 0.010	−0.715	0.367	0.430
pH 6.0, 4.16 mg/L oxygen	−0.694 ± 0.008	0.377 ± 0.004	0.443 ± 0.005	−0.715	0.377	0.443
pH 5.0, 4.11 mg/L oxygen	−0.729 ± 0.005	0.337 ± 0.012	0.396 ± 0.014	−0.713	0.397	0.466
